# Financial toxicity and distress among prostate cancer patients in Lebanon: a cross-sectional study

**DOI:** 10.3389/fpubh.2026.1797663

**Published:** 2026-08-04

**Authors:** Michel Abi Karam, Anthony Mina, Elie Ghadban, Ziad Azar, Jean Khalil, Ghida El Khoury, Sabine El Breidy, Charbel El Hachem, Rodrigue Saad, Antoine Kassis, Raghid El Khoury

**Affiliations:** 1 School of Medicine and Medical Sciences, Holy Spirit University of Kaslik, Jounieh, Lebanon; 2 Urology Department, Notre Dame des Secours University Hospital, Byblos, Lebanon

**Keywords:** economic crisis, FACIT-COST, financial toxicity, health insurance, Lebanon, PHQ-4, prostate cancer, psychological distress

## Abstract

**Background:**

Financial toxicity is a major component of the cancer experience. Prostate cancer often requires prolonged treatment and follow-up, increasing financial and psychological burden. Lebanon's economic crisis has disrupted health-care financing and insurance coverage, potentially worsening these burdens. This study evaluated financial toxicity among male patients with prostate cancer in Lebanon and its relationship with psychological distress.

**Methods:**

We conducted a cross-sectional telephone survey of adult males (≥18 years) with confirmed prostate cancer receiving active treatment or with prior prostate surgery. Validated Arabic Functional Assessment of Chronic Illness Therapy–Cost (FACIT–COST) and Patient Health Questionnaire-4 (PHQ−4) were administered, and sociodemographic, financial, and clinical data were obtained from medical records and self-report. Linear regression assessed associations with financial toxicity and psychological distress, and a multivariate model evaluated their relationship while controlling for other variables.

**Results:**

We enrolled 147 patients (mean age 71.8 ± 8.3 years). Limited treatment coverage, treatment disruptions, and lower household income were independently associated with higher financial toxicity. Treatment disruptions and lower household income were associated with higher psychological distress. Financial toxicity was significantly associated with psychological distress (β = −0.21 per unit increase in FACIT–COST; *p* < 0.001), and this association was not explained by clinical or sociodemographic factors.

**Conclusion:**

Financial toxicity is prevalent among men with prostate cancer in Lebanon and is strongly associated with psychological distress. Household income and treatment continuity appear more influential than disease severity, highlighting the need for economic support systems and stable insurance coverage in crisis-affected settings.

## Introduction

Prostate cancer is the most frequent cancer in men globally, with a high prevalence among aging populations (1). Recent advancements in screening and treatment modalities have resulted in increased incidence and improved survival rates for prostate cancer patients (2). This results in longer treatment durations and follow up due to patients surviving longer. Thus, prostate cancer is considered a chronic long-term illness with a prolonged disease trajectory in many patients. Effectively managing and treating this disease requires a broad multidisciplinary medical treatment plan. Also, the treatment options available to patients are variable case by case (local treatments; systemic treatments; combinations of both) with potential severe long-term side effects taken into consideration (3). Some studies suggest that patients undergoing treatment for prostate cancer reported decreased life satisfaction and increased levels of anxiety and depression compared to the general population (4). Accordingly, more and more evidence indicates that the global burden of prostate cancer morbidity is increasing, necessitating urgent public health interventions (5).

Financial toxicity concept has been developed in order to effectively quantify the financial distress caused by diagnosis and treatment in cancer patients as direct and indirect costs accumulate. Financial toxicity is now widely considered a significant and measurable burden of cancer care, with consequences comparable to other treatment-related adverse effects (6). It has become a valid and reliable way to assess the implications of financial distress and strain perceived by patients (6). An increasing quantity of evidence indicates that increased financial toxicity is associated with reduced quality of life, excess mortality and worse psychological distress (7, 8). As a result, it has become essential to take into consideration and monitor this integral element of cancer survivorship with many implications for clinical outcomes.

The age standardized incidence rate of prostate cancer was at its peak in Lebanon in 2012 and exceeded the global average incidence rate for that same year (9). Furthermore, Lebanon's healthcare system is challenged in collecting reliable cancer registry and epidemiology data, hindering their ability to implement effective public health interventions (10). Lebanon is presently facing an unprecedented economic crisis due to the COVID-19 pandemic, and continued sociopolitical instability. This turmoil has severely disrupted the healthcare system, leading to critical shortages of medications and treatments (11, 12). In fact, patients during the economic crisis in Lebanon had levels of insurance limitations with many patients failing to get insurance coverage for their treatment regarding their current insured status. Essential drugs are often unavailable, prohibitively expensive, or only accessible through the black market, leaving patients without reliable access to necessary care (13, 14). The already challenging journey of cancer treatment is exacerbated by the scarcity of medical resources and financial constraints, making it difficult for patients to adhere to prescribed therapies and manage side effects effectively (13–15). Moreover, the stress and uncertainty associated with the broader socio-economic crisis further contribute to the deterioration of their mental health and health-related quality of life (HRQoL) (16).

The main purpose of this study was to examine the relationship among a variety of socioeconomic and clinical factors in individuals diagnosed with prostate cancer with experiencing financial toxicity during the ongoing economic crisis in Lebanon. The secondary objectives were to analyze the relationship between those factors as they relate to levels of psychological distress; and also to evaluate the relationship between financial toxicity and psychological distress. It was hypothesized that individuals with poorer socioeconomic status and worse clinical status would have higher levels of financial toxicity and subsequently higher levels of psychological distress. In doing so, it is expected to identify which interventions may be most effective in reducing the financial toxicity on this high-risk population, and assist in improving their adherence to treatments and optimizing health-related outcomes at this time when there are many challenges facing them.

## Methods

### Study design and setting

The study was conducted between January 2025 and July 2025, as a cross- section, observational design; at one monocenter, private hospital in Lebanon. Adult Prostate Cancer patients that were being treated at the time of the study were recruited from the center. A structured questionnaire was used to collect data for this study. The questionnaires were administered via phone by members of the research team. The research team decided to administer the questionnaires via phone to assure accuracy of the data that was collected and to provide an opportunity for the patients to participate in the study during the current, ongoing economic crisis in Lebanon, which has negatively impacted the ability of patients to access care and for patients to be followed post- surgery (17).

### Study population and participants

Eligible participants were Lebanese adult patients aged 18 years or older with a documented and histologically confirmed diagnosis of prostate cancer (any Gleason stage), who were either receiving or having received any prostate cancer specific treatment (surgery, radiotherapy, androgen deprivation therapy, chemotherapy, etc.) at “Notre Dame des Secours University Hospital (NDSUH)” a tertiary care center between February 2020 and July 2025. Patients with cognitive impairment, inability to complete the telephone interview, or those who declined participation were excluded. Cognitive impairment was determined based on medical record documentation or inability to provide informed consent or reliably complete the telephone questionnaire. Exclusion criteria included patients with previous psychiatric diagnosis of depression or anxiety.

Participants were recruited using urology clinic lists and medical records from the NDSUH care center. Eligible patients were contacted by a non-treating physician member of the research team and invited them to participate to the telephone data collection. A maximum of three contact attempts was made, after which individuals were considered non-responders.

A total of 232 patients were contacted, 12 did not participate and 21 had to be excluded due to cognitive impairments that would have made it impossible for them to complete the telephone interview. There were 52 other patients who were excluded due to non-response. In total 147 patients were included in the final analysis. Consecutive sampling was used.

### Minimal sample size

Using G^*^Power version 3.1 (Heinrich-Heine-Universität Düsseldorf, Düsseldorf, Germany), we calculated the minimal sample size to determine the relationship between the predictor variables and the outcome variable using the Linear Multiple Regression model. We assumed a medium effect size (*f*^2^=.15) according to Cohen guidelines, a *p*-value (alpha) of 0.05, an 80% power to detect statistically significant relationships, and 14 predictor variables. The obtained minimum sample size is 135 participants. Therefore, our final study sample is larger than the minimum required sample size.

### Study data collection procedures

We collected data via a structured, telephone administered survey, developed by a non-treating physician member of the research team. We developed a standardized script to insure that the same questions and responses were asked and received by each participant. The questionnaire was administered in Arabic, and each interview lasted approximately 10 to 20 min.

Data regarding clinical variables such as treatment-related and surgical information, were extracted from medical records and subsequently confirmed by the interviewer during the telephone call in order to enhance data accuracy. Data completeness was assessed immediately following each interview, and no missing data were recorded.

### Study variables

The primary outcome variable was financial toxicity, assessed using the Functional Assessment of Chronic Illness Therapy–Cost (FACIT-COST) total score and analyzed as a continuous variable. It is one of the most widely used scales to assess financial toxicity in cancer patients (6). Psychological distress was assessed using the Patient Health Questionnaire-4 (PHQ-4) total score. PHQ-4 is also a widely used scale designed to measure general psychological distress by screening for symptoms of anxiety and depression (18). Psychological distress was considered a secondary outcome and it was also analyzed as a continuous variable.

Sociodemographic variables collected from patients included age, location of residence, education level, and marital status. Financial and socioeconomic variables included the status healthcare insurance coverage (non-insured, public insurance, private insurance), effective of quality of financial coverage by the insurance (no financial coverage, partial cost coverage, total cost coverage), average monthly household income (low, medium, and high), financial details related to treatment payment, and whether prescribed medications were substituted and purchased from another country. Treatment cost coverage referred to the proportion of treatment-related costs actually borne by the patient's insurance at the time of care, as reported by the patient, and was categorized as full, partial, or no coverage; this was distinguished from insurance enrollment status (non-insured, public, and private). Treatment disruption referred to any deviation from the planned treatment schedule and was classified as a delay (treatment postponed beyond its scheduled date but ultimately received) or an interruption (complete discontinuation of a planned treatment). Patients with neither were classified as having no disruption. Household income was categorized as low (under 300 USD per month), medium (from 300 to 1,000 USD per month), and high (above than 1,000 USD per month) according to classifications previously employed in Lebanese studies conducted during the ongoing economic crisis (14).

Clinical variables included prostate cancer grade and aggressiveness using Gleason score. Gleason score was considered low risk, scores of 7 were considered intermediate and scores of 8 and higher were considered high risk (19). Another clinical variable was current prostate cancer treatment strategy. One category of patients was undergoing local treatment strategies such as surgery and radiotherapy. Other patients were undergoing systemic therapy such as androgen deprivation therapy or chemotherapy. The third category of patients was following a combined treatment regimen. Presence of other comorbidities, treatment delays and their duration, and if complete interruption of treatment occurred were also taken into consideration. Participant names were collected for identification and contact purposes during data collection and were not included in the analysis.

### Measurement instruments

Financial toxicity was assessed using Comprehensive Score for Financial Toxicity version 2 (FACIT-COST). It is a validated scale designed to evaluate the financial toxicity associated with chronic illness (6). The Arabic version of the FACIT-COST was used in this study. This scale has been previously validated for the use in Arab speaking cancer patients (20). This scale is comprised of 12 items with each item scored by a Likert scale (0 to 4). One of the 12 items is a summary item which was not scored or included in total score calculation. FACIT-COST score was calculated according to the instructions published by the FACIT group. FACIT-COST score was analyzed as a continuous variable. Total score range is 0 to 44. Higher total scores indicate better financial well-being and less financial toxicity.

Psychological distress was assessed using the Patient Health Questionnaire-4 (PHQ-4). It consists of a four item Likert scale questionnaire (0–3). Two items assess depression and two others assess anxiety. The Arabic version of the PHQ-4 was previously validated (21). Total score was calculated and was used as a continuous measure of psychological distress. Total score range is 0 to 12.

Both questionnaires were read verbatim to participants during the telephone interviews. When needed, clarification was provided in case patients needed it. All questionnaire items were completed, resulting in no missing data.

### Ethical considerations

The study protocol was reviewed and approved by the ethics committee of Notre Dame des Secours University Hospital ethics committee. Verbal informed consent was obtained from all participants prior to data collection. Participation was voluntary, and confidentiality of participant information was strictly maintained throughout the study. All aspects of the study adhered to the ethical principles of research (e.g., voluntary participation, confidentiality of participants, disclosure of the purpose and methods of the study).

### Statistical analysis

Using R statistical software version 4.5.1 (R Foundation for Statistical Computing, Vienna, Austria) statistical analysis was conducted to assess the associations between sociodemographic, clinical and financial characteristics of prostate cancer patients and their levels of financial toxicity (total FACIT-COST score), and psychological distress (total PHQ-4 score).

Descriptive statistics were first used to describe our sample, including frequency counts and percent distributions of all categorical variables, and mean and standard deviation estimates for all continuous variables.

Multiple linear regression models were then developed to examine the association between each variable, and each of the dependent variables. The selection of predictor variables to include in each model were determined a priori, based upon clinical importance. Therefore, age, was entered into each model as a continuous variable, and all categorical predictor variables had an appropriate reference group for comparison.

Three multivariable models were constructed to map directly onto the study's three objectives. Model 1 identified factors independently associated with financial toxicity (primary objective). Model 2 identified factors associated with psychological distress (secondary objective). Model 3 added the FACIT-COST score to the distress model to test whether financial toxicity was independently associated with psychological distress after accounting for the same covariates. Comparing Models 2 and 3 was intended to clarify whether socioeconomic and treatment-related predictors of distress operated independently of financial toxicity or were attenuated once it was included.

In addition to determining which predictor variables to enter into the models, it is also necessary to determine if the assumptions required for linear regression are met. These assumptions include linearity, normality of residuals and homogeneity of variance. In order to determine whether these assumptions have been violated we visually inspected residual diagnostic plots that are provided with every linear regression model (22). Residual-versus-fitted, scale-location, and normal Q–Q plots for each model are provided as Supplementary Figure S1.

For this analysis, we found that none of the assumptions have been violated and therefore the models can be interpreted as indicating valid relationships between the predictor variables and the dependent variables. Additionally, multicollinearity was examined through evaluation of variance inflation factor (VIF) values for all of the predictor variables in the models (23). Because the models included categorical predictors, multicollinearity was assessed using generalized variance inflation factors (GVIF) and their degrees-of-freedom-adjusted equivalents (GVIF^∧^[1/(2·Df)]). All adjusted values were below 1.6, well under the conventional threshold of 5, indicating that multicollinearity was not a concern. Full GVIF values for each model are provided as Supplementary Material Table S1. Statistical significance was determined to be two tail and less than 0.05. For each model, regression coefficients and *p*-values were reported. There were no missing data points in this dataset.

## Results

The study sample consisted of 147 men diagnosed with prostate cancer. Participant characteristics are summarized in Table 1. The average age of the sample was 71.8 years (SD = 8.25). The average FACIT-COST score was 14.6 (SD = 10.7); the average PHQ-4 score was 5.16 (SD = 3.95).

**Table 1:** Sociodemographic, clinical, and financial characteristics of the study population (*N* = 147).

Variable	*n* (%) or Mean ±SD
Age (years)	71.8 ± 8.25
FACIT-COST score	14.6 ± 10.7
PHQ-4 score	5.16 ± 3.95
Gleason risk group	
Low (Gleason score = 6)	8 (5.4%)
Intermediate (Gleason score = 7)	74 (50.3%)
High (Gleason score= 8; 9; 10)	65 (44.2%)
Healthcare insurance type	
Non-insured	20 (13.6%)
Private insurance	45 (30.6%)
Public insurance	82 (55.8%)
Treatment modality	
Local treatment only	57 (38.8%)
Systemic treatment only	38 (25.9%)
Combined treatment	52 (35.4%)
Insurance coverage for treatment	
Full payment coverage	38 (25.9%)
Partial payment coverage	66 (44.9%)
No coverage whatsoever	43 (29.3%)
Treatment disruption	
None	90 (61.2%)
Delay	46 (31.3%)
Interruption	11 (7.5%)
Monthly household income	
Low	53 (36.1%)
Medium	59 (40.1%)
High	35 (23.8%)
Comorbidities	
No	45 (30.6%)
Yes	102 (69.4%)

Concerning clinical aspects of prostate cancer, most participants (50.3%) were classified as having an intermediate Gleason score (4+3; 3+4) or as having a high-risk classification for prostate cancer (44.2%) based on their Gleason score (e.g., 8, 9, 10), whereas a relatively small number of participants were classified as having low risk disease (Gleason score = 6; 5.4%). Most participants received either local treatment only (38.8%), systemic treatment only (25.9%) or both local and systemic treatments (35.4%). A significant number of participants experienced some presence of comorbidity (69.4%).

Regarding financial/insurance factors, over half of the participants were covered under public insurance programs (55.8%), approximately one-third were covered through private insurance (30.6%), and approximately one in seven were non-insured (13.6%). The actual level of insurance coverage for treatment was also variable among participants, with almost 26 percent reporting that their insurance covered all costs associated with treatment, 45 percent reporting that their insurance provided partial coverage of treatment-related expenses, and nearly 30 percent reporting that their insurance provided absolutely no coverage for treatment. Finally, 39 percent of participants reported experiencing treatment disruptions, which included delays in receiving treatment (31.3%) and discontinuation of treatment (7.5%). Monthly household income was reported as low in 36.1% of participants, medium in 40.1%, and high in 23.8%.

Unadjusted (bivariate) linear regression analyses were performed, and β coefficients for the unadjusted associations are presented in Table 2. Adjusted associations are shown in multivariable models.

**Table 2:** Unadjusted (bivariate) linear regression analyses of factors associated with financial toxicity (FACIT-COST) and psychological distress (PHQ-4).

Predictor	Category (vs. reference)	FACIT-COST β	*p*-value	PHQ-4 β	*p*-value
Age (years)	Per 1-year increase	0.05	0.64	−0.01	0.87
Gleason risk group	Intermediate vs. High	5.59	0.002	−1.92	0.004
	Low vs. High	8.54	0.029	−1.86	0.202
Insurance type	Private vs. No coverage	7.36	0.008	−1.20	0.254
	Public vs. No coverage	0.39	0.88	0.59	0.54
Treatment modality	Local vs. Combined	5.29	0.008	−1.55	0.039
	Systemic only vs. Combined	−2.72	0.22	0.28	0.74
Actual treatment coverage	None vs. Full	−12.00	< 0.001	2.11	0.016
	Partial vs. Full	−8.85	< 0.001	2.04	0.011
Treatment disruption	Interruption vs. Delay	2.87	0.33	−1.11	0.33
	None vs. Delay	13.40	< 0.001	−4.53	< 0.001
Monthly household income	Low vs. High	−17.70	< 0.001	5.51	< 0.001
	Medium vs. High	−7.57	< 0.001	2.22	0.002
Comorbidities	Yes vs. No	−0.81	0.68	0.56	0.43

(FACIT-COST and PHQ-4 scores analyzed as continuous outcomes. β coefficients represent unadjusted associations relative to reference categories.) FACIT-COST, functional assessment of chronic illness therapy–cost; PHQ-4, patient health questionnaire-4.

Multivariable linear regression analysis was performed to examine factors independently associated with financial toxicity, measured by the FACIT-COST total score (Table 3). After adjustment for sociodemographic, financial, and clinical variables, lower levels of actual treatment coverage, treatment disruption, and lower monthly household income were significantly associated with worse financial toxicity in comparison to the reference categories. Other variables, including age, Gleason risk group, treatment modality, insurance type, and comorbidities, were not significantly associated with FACIT-COST scores.

**Table 3:** Model 1: Multivariable linear regression analysis for financial toxicity (FACIT-COST score).

Predictor	β (Estimate)	95% CI	*p*-value
Age (years)	−0.02	−0.17 to 0.13	0.774
Comorbidities (Yes vs No)	0.40	−2.16 to 2.95	0.759
Gleason risk group			
Intermediate (low)	−1.68	−7.08 to 3.72	0.540
High (low)	−3.09	−8.91 to 2.74	0.297
Insurance type			
Non-insured (private)	−3.98	−8.38 to 0.42	0.076
Public (private)	−0.50	−3.56 to 2.56	0.747
Treatment modality			
Combined (local)	0.95	−2.23 to 4.14	0.554
Systemic only (local)	2.22	−1.44 to 5.87	0.232
Actual treatment coverage			
None (full)	−5.75	−9.69 to −1.80	**0.005**
Partial (full)	−4.53	−7.88 to −1.19	**0.008**
Treatment disruption			
Delay (none)	−7.33	−10.58 to −4.07	**< 0.001**
Interruption (none)	−4.42	−9.31 to 0.46	0.076
Monthly household income			
Low (high)	−13.73	−17.39 to −10.07	**< 0.001**
Medium (high)	−6.70	−9.90 to −3.49	**< 0.001**

Outcome: FACIT-COST total score (continuous). Reference categories are in parentheses.

Multivariable linear regression analysis was conducted to identify factors independently associated with psychological distress, measured by the PHQ-4 total score (Table 4). After adjustment for sociodemographic, financial, and clinical variables, treatment disruption and lower monthly household income were significantly associated with higher psychological distress scores. Other variables, including age, Gleason risk group, insurance type, treatment modality, actual treatment coverage, and comorbidities, were not significantly associated with PHQ-4 scores.

**Table 4:** Model 2: Multivariable linear regression analysis for psychological distress (PHQ-4 score).

Predictor	β (Estimate)	95% CI	*p*-value
Age (years)	0.03	−0.04 to 0.09	0.454
Comorbidities (yes vs. no)	0.26	−0.86 to 1.38	0.650
Gleason risk group			
Intermediate (low)	0.05	−2.33 to 2.42	0.969
High (low)	1.06	−1.50 to 3.61	0.416
Insurance type			
Non-insured (private)	1.42	−0.51 to 3.35	0.148
Public (private)	0.24	−1.11 to 1.58	0.729
Treatment modality			
Combined (local)	−0.65	−2.05 to 0.75	0.358
Systemic only (local)	−1.56	−3.16 to 0.05	0.057
Actual treatment coverage			
None (full)	−0.44	−2.17 to 1.30	0.620
Partial (full)	0.41	−1.06 to 1.88	0.578
Treatment disruption			
Delay (none)	3.58	2.15 to 5.01	**< 0.001**
Interruption (none)	2.36	0.22 to 4.51	**0.031**
Monthly household income			
Low (high)	3.85	2.24 to 5.46	**< 0.001**
Medium (high)	1.55	0.14 to 2.96	**0.031**

Outcome: PHQ-4 total score (continuous). Reference categories are shown in parentheses.

An additional multivariable linear regression model was conducted to examine the association between financial toxicity and psychological distress (Table 5). After adjustment for sociodemographic, financial, and clinical variables, lower FACIT-COST scores were independently associated with higher PHQ-4 scores. Treatment delays and lack of treatment coverage were also associated with increased psychological distress, while other covariates were not significantly associated with PHQ-4 scores.

**Table 5:** Model 3: Multivariable linear regression analysis examining the association between financial toxicity and psychological distress (PHQ-4 score).

Predictor	β (Estimate)	95% CI	*p*-value
FACIT-COST score	−0.21	−0.27 to −0.14	**< 0.001**
Age (years)	0.02	−0.04 to 0.08	0.488
Comorbidities (yes vs. no)	0.34	−0.65 to 1.33	0.499
Gleason risk group			
Intermediate (low)	−0.30	−2.40 to 1.80	0.776
High (low)	0.41	−1.86 to 2.69	0.719
Insurance type			
Non-insured (private)	0.59	−1.14 to 2.32	0.498
Public (private)	0.13	−1.06 to 1.32	0.826
Treatment modality			
Combined (local)	−0.45	−1.69 to 0.78	0.469
Systemic only (local)	−1.10	−2.52 to 0.33	0.131
Actual treatment coverage			
None (full)	−1.63	−3.21 to −0.05	**0.043**
Partial (full)	−0.53	−1.86 to 0.81	0.434
Treatment disruption			
Delay (none)	2.05	0.70 to 3.41	**0.003**
Interruption (none)	1.44	−0.48 to 3.36	0.140
Monthly household income			
Low (high)	1.00	−0.70 to 2.69	0.246
Medium (high)	0.16	−1.17 to 1.48	0.814

Outcome: PHQ-4 total score (continuous). Reference categories are shown in parentheses.

## Discussion

The aim of this cross-sectional study was to investigate the association between many socioeconomic factors (age, insurance status, degree of coverage of treatments by insurance, household income) and clinical factors (Gleason score, current treatment approach, treatment disruption, and comorbidities) with financial toxicity and psychological distress in Lebanese prostate cancer patients during the economic crisis that began in end of 2019 (24). The findings suggest the presence of an important financial and psychological distress in this population and emphasize the role of treatment affordability, income level, and treatment access and continuity as factors associated with patient outcomes.

Patients with limitations from their insurance coverage and payment (no coverage and partial coverage of treatments) were independently associated with worse financial toxicity in comparison to patients with full medical expense coverage by insurance. This effect was found after adjustment for other variables such as insurance type, family income, and clinical factors. This finding suggests that in the context of economic instability, the insurance status alone does not adequately represent the financial toxicity experienced by patients. This is due to the worsening quality of insurance coverage during this period. In Lebanon, the process of insurance compensation was totally altered and disrupted as a result of the economic crisis (11). In practice, this resulted in reduced effective insurance protection for the patients, and increased out-of-pocket expenses. This may explain the finding that status of the coverage of treatment by insurance came across as a strong and statistically significant determinant of financial toxicity and that insurance status by itself was not. These results show the importance of evaluating the real-world coverage in every patient rather than insurance status alone when assessing financial toxicity in crisis settings.

For the treatment disruption variable, a significant association was found with financial toxicity and with psychological distress. Notably, patients who experienced delays in treatment reported significantly worse financial toxicity and significantly worse psychological distress. Also, patients with complete treatment interruption reported significantly worse psychological distress. These worse outcomes highlight the compounding effect of the lack of treatment continuity during the economic crisis. Adherence to prostate cancer treatment is essential in ensuring adequate outcomes and to prevent disease progression (25). It is even more critical to avoid delays in the setting of patients taking systemic therapy (hormonotherapy) in order to achieve an optimal clinical outcome and maximize survival. However, Lebanon's current economic crisis has further exacerbated healthcare challenges, including the availability and accessibility of treatments, which in turn, compromise patient adherence (26, 27). Accordingly, treatment disruptions in cancer care may exacerbate psychological distress due to the additional uncertainty, and fear of disease progression that patients have to deal with. This strong association between treatment disruptions and PHQ-4 scores stresses the importance of treatment continuity on psychological distress.

Family household income demonstrated a strong and significant association with financial toxicity as well as psychological distress. In comparison to high income households (reference group), patients from low- and medium-income households both experienced considerably worse financial toxicity and higher psychological distress. However, the lower income household group had stronger associations. Regarding financial toxicity, low-income household factor was more strongly associated with a greater financial toxicity increase compared with the medium household income category. These findings align with the current literature regarding the association of lower income categories with higher financial toxicity (28). Also lower income patients may be more vulnerable during economic crisis due to being less able to adapt when facing new unexpected out-of-pocket cancer related expenses. Similar findings were noted regarding psychological distress having a stronger association with the lower household income compared to the medium income category. These results highlighting the strong association between household income and financial toxicity as well as PHQ-4 scores stress the importance of having better economic safety nets beyond healthcare insurance in the setting of economic instability.

In addition to the independent socioeconomic and healthcare-related variables, our findings suggest that financial toxicity score itself was independently linked with psychological distress after adjustment for income, treatment disruption, coverage, and clinical characteristics. Having higher financial toxicity (lower FACIT-COST score) was associated with higher psychological distress (PHQ-4 score). The financial toxicity experienced by patients may act as a psychological stress factor in prostate cancer patients associated with more anxiety and depression. This finding suggests that financial toxicity represents a burden by itself which may exacerbate psychological distress regardless of the baseline clinical factors and socioeconomical status of the patient.

Notably, the associations of lower household income and treatment disruption with psychological distress were substantially attenuated once financial toxicity was added to the model (Table 5), whereas financial toxicity itself remained strongly associated with distress. This pattern is consistent with financial toxicity lying on the pathway between socioeconomic adversity and psychological distress, potentially acting as an intermediary. Given the cross-sectional design, however, this interpretation is hypothesis-generating only and cannot establish mediation or temporal ordering.

During economic crisis conditions, patients frequently have to deal with uncertainty regarding treatment availability, continuity and insurance compensation for expenses. The independent association between financial toxicity and psychological distress highlights the importance of taking into consideration financial concerns in order to ensure comprehensive and adequate cancer management for patients. Practices that ensure minimal financial toxicity such as improved insurance coverage, may be associated with reduced psychological distress that is independently associated with it. This is essential to consider during economic crisis such as the one happening in Lebanon since 2019.

In contrast, some other variables such as age, Gleason risk group, treatment modality, insurance type, the presence of any comorbidities were not found to be independently associated with financial toxicity or psychological distress after adjustment. Within this economic crisis environment, insurance coverage, household income and predominated other clinical, demographic and socioeconomic variables in their association with financial toxicity and psychological distress. Current evidence shows an association between patients aged under 65 years and higher financial toxicity scores (29). The absence of association between these two variables in our findings may be caused by our relatively older patient group. Further, evidence states that more aggressive prostate cancer forms are associated with higher financial toxicity (30). This association was not observed in the current study. The context of Lebanon's economic crisis may have resulted in financial factors taking priority in influencing the patients' financial experience.

Several limitations should be considered when interpreting these results. The cross-sectional design cannot infer causality and cannot evaluate the temporal relationships between financial toxicity, psychological distress, and treatment disruption. In addition, patients were assessed at varying points in their treatment course (some during active therapy and others after completion) and financial and disruption data were partly self-reported. This heterogeneity in timing may introduce recall bias, as patients reporting retrospectively may estimate past costs and disruptions less accurately than those currently in treatment. The study was conducted at a single private tertiary care center, which limits generalizability. Patients at private institutions may differ systematically from those treated in public hospitals or NGO-supported facilities—for example, in insurance mix, baseline socioeconomic status, and out-of-pocket exposure—so the magnitude of financial toxicity reported here may not reflect that of underserved or publicly treated populations, in whom it could plausibly be greater. In addition, the telephone-based design may have underrepresented patients without reliable phone access, a group potentially more economically vulnerable. The generalizability of the study's results to other patient populations in various healthcare environments could be limited as a result of potential biases such as non-response and sampling bias. Although the final sample exceeded the minimum required by the a priori power calculation, the ratio of predictors to observations was modest, which is reflected in the relatively wide confidence intervals for some estimates and warrants cautious interpretation of the less precise coefficients.

Although the PHQ-4 is a screening tool for assessing psychological distress, it does not serve as a full diagnostic tool; however, the study's use of validated Arabic scales and interviewer data collection supports the internal validity of this research.

Regardless of its limitations, this research represents an important contribution to our understanding of the multifaceted burden experienced by prostate cancer patients within the setting of a low and middle income country that is experiencing a financial collapse. The findings also emphasize the need for policy interventions that will go beyond the simple process of enrolling in health insurance to provide meaningful treatment coverage, to prevent disruptions in care and to provide targeted financial and psychosocial support to economically vulnerable patients. In addition, addressing financial toxicity associated factors represents one of the most direct routes to improve both financial and mental health status of cancer patients in settings that are experiencing economic crises. By controlling for confounders, we were able to make the results as accurate and reliable as possible.

## Conclusion

The quality of life of prostate cancer patients has declined significantly since the economic crisis began in Lebanon because they have had to endure significant levels of financial toxicity and psychological distress. According to this research, several factors including treatment interruption, inadequate coverage of treatment costs and lower household incomes are associated with higher financial toxicity and greater psychological distress. Therefore, maintaining the ability of insurance programs to act as a safety net for patients to provide them with protection against the risks and uncertainties inherent in seeking medical treatment, particularly in times of economic uncertainty may be associated with better overall well-being among cancer patients, though this hypothesis requires longitudinal confirmation.

## Data Availability

The raw data supporting the conclusions of this article will be made available by the authors, without undue reservation.
