# Supplementary material for: Financial toxicity and distress among prostate cancer patients in Lebanon: a cross-sectional study
Source: Front Public Health. 2026 Aug 4;14:1797663. doi: 10.3389/fpubh.2026.1797663 (PMC13481321; doi:10.3389/fpubh.2026.1797663)
Supplement: Supplementary file 1 [file Image_1.pdf]

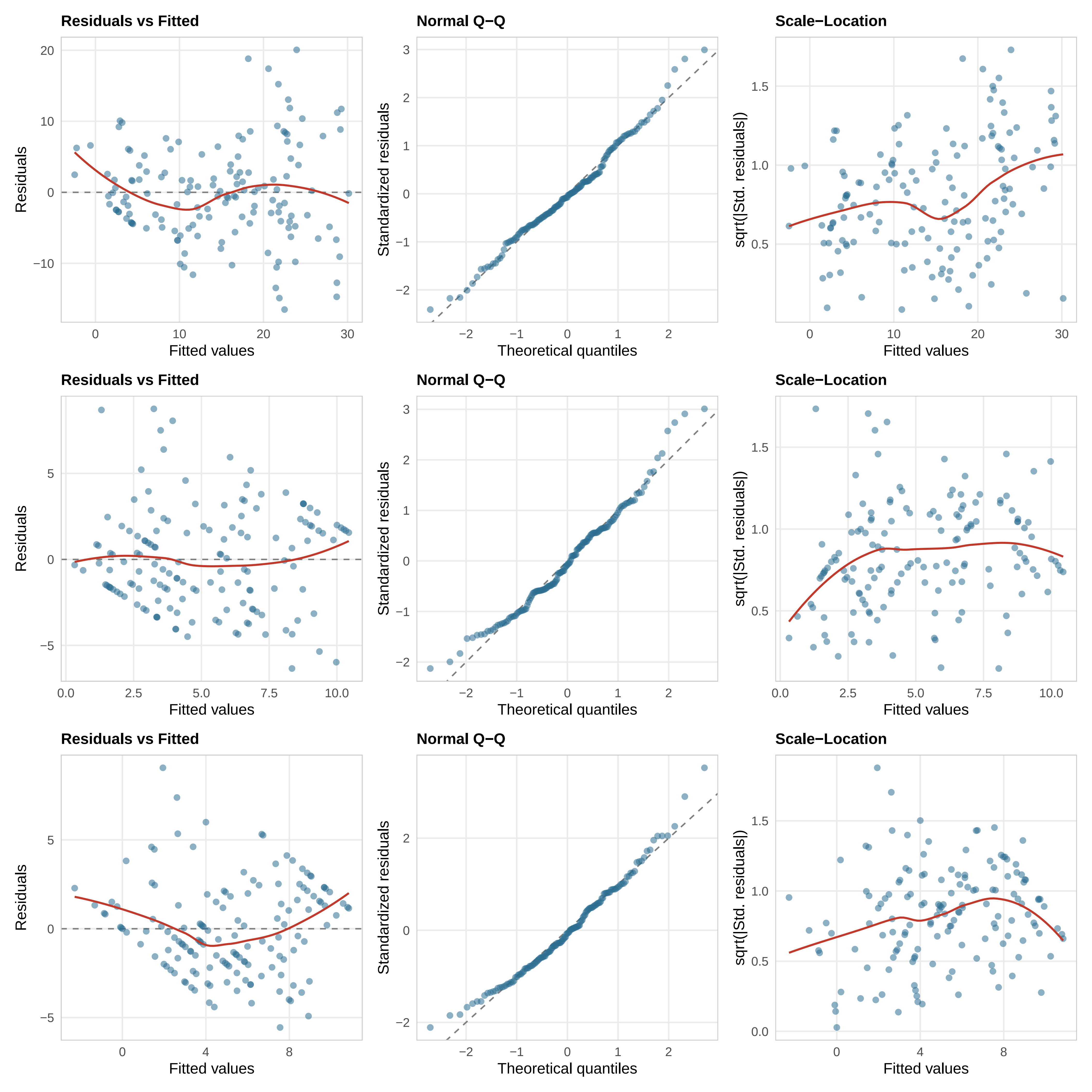

Residuals vs Fitted
Residuals
Fitted values
Normal Q−Q
Standardized residuals
Theoretical quantiles
Scale−Location
sqrt(|Std. residuals|)
Fitted values
Residuals vs Fitted
Residuals
Fitted values
Normal Q−Q
Standardized residuals
Theoretical quantiles
Scale−Location
sqrt(|Std. residuals|)
Fitted values
Residuals vs Fitted
Residuals
Fitted values
Normal Q−Q
Standardized residuals
Theoretical quantiles
Scale−Location
sqrt(|Std. residuals|)
Fitted values
