# Supplementary material for: Financial toxicity and distress among prostate cancer patients in Lebanon: a cross-sectional study
Source: Front Public Health. 2026 Aug 4;14:1797663. doi: 10.3389/fpubh.2026.1797663 (PMC13481321; doi:10.3389/fpubh.2026.1797663)
Supplement: Supplementary file 2 [file Table_1.pdf]

Supplementary Material Table S1. Multicollinearity diagnostics (GVIF and adjusted GVIF) for the three multivariable regression models.

Predictor	Df	Models 1 & 2		Model 3	
		GVIF	Adj. GVIF	GVIF	Adj. GVIF
FACIT-COST score	1	—	—	2.54	1.59
Age	1	1.18	1.09	1.19	1.09
Gleason risk group	2	1.50	1.11	1.52	1.11
Insurance	2	1.69	1.14	1.74	1.15
Treatment	2	1.87	1.17	1.89	1.17
Coverage	2	1.93	1.18	2.06	1.20
Treatment disruption	2	1.84	1.17	2.12	1.21
Household income	2	1.63	1.13	2.32	1.23
Comorbidities	1	1.05	1.02	1.05	1.02

Abbreviations: FACIT-COST, Functional Assessment of Chronic Illness Therapy–Comprehensive Score for financial Toxicity; GVIF, generalized variance inflation factor; Df, degrees of freedom.
